# Fuzzy Planar Cell Polarity Gene (FUZ) Promtes Cell Glycolysis, Migration, and Invasion in Non-small Cell Lung Cancer via the Phosphoinositide 3-Kinase/Protein Kinase B Pathway

**DOI:** 10.7150/jca.63152

**Published:** 2022-05-01

**Authors:** Shu Wang, Hui Zhang, Bulin Du, Xuena Li, Yaming Li

**Affiliations:** Department of Nuclear Medicine, The First Hospital of China Medical University, Shenyang, P.R.China; Shu Wang and Hui Zhang have contributed equally to this work and share first authorship

**Keywords:** FUZ, NSCLC, glycolysis, migration, invasion

## Abstract

**Purpose:** Fuzzy planar cell polarity gene (FUZ) is regarded as a planar cell polarity effector that controls multiple cellular processes during vertebrate development. The role of FUZ in glucose metabolism, invasion, and metastasis of non-small cell lung cancer (NSCLC) is unclear. The aims of this study were to investigate the relationship between FUZ and glucose metabolism and its mechanism of action.

**Materials and methods:** Quantitative real-time polymerase chain reaction (qRT-PCR) analysis was used to detect FUZ expression in A549 and H1299 cells. Additionally, qRT-PCR and western blot analysis were used to detect the expression of related glucose metabolism indicators, and lactate and 18 Fluorine fludeoxyglucose (^18^F-FDG) uptake assays used to detect changes in glucose metabolites. Further, cell invasion and migration behavior were evaluated by transwell and wound healing assays. In vivo tumor growth assay was conducted to assess the effect of FUZ.

**Results:** We found that FUZ was significantly upregulated in the NSCLC cell lines compared to that in the normal HBE cells. FUZ was found to promote energy metabolism through the phosphoinositide 3-kinase (PI3K)/protein kinase B (AKT) pathway, and overexpression of FUZ increased both lactic acid and ^18^F-FDG uptake. Moreover, FUZ knockdown significantly inhibited the migration and invasion of NSCLC cells. In vivo, FUZ knockdown can significantly inhibit tumor proliferation in the xenograft model, which was well identified by Micro-PET scan.

**Conclusion:** The present finding in vitro and vivo show that FUZ is involved in NSCLC cell energy metabolism, invasion and migration via the PI3K/AKT signaling pathway, suggesting that FUZ can be a potential therapeutic target for NSCLC.

## 1. Introduction

Lung cancer is a leading cause of cancer-related deaths worldwide, and non-small cell lung cancer (NSCLC), which is the main subtype of lung cancer, accounts for 85% of all lung cancer cases 1. NSCLC is commonly treated using surgery, chemotherapy, and targeted therapies; however, due to its characteristic rapid progression, ease of metastasis, and chemoresistance, the 5-year survival rates for patients with NSCLC remain poor 2. Hence, new treatment strategies based on novel molecular targets are urgently required to improve the prognosis of patients with NSCLC.

Aberrant energy metabolism is an important characteristic of tumors 3. Energy metabolism reprogramming (EMR), a newly discovered hallmark of cancer cells, is essential for the survival of cancer cells and can increase their proliferation, migration, and invasion capacities 4. Hexokinase 2 (HK2), pyruvate kinase 2 (PKM2), and lactate dehydrogenase A (LDHA) can regulate EMR, and abnormal expression of these enzymes may promote the Warburg effect in cancer cells 5-7. Phosphoinositide 3-kinase (PI3K)/protein kinase B (AKT) signaling plays an important role in maintaining glucose homeostasis and is a key pathway in tumor energy metabolism, where it inhibits glycolysis in malignant cells 8,9.

Fuzzy planar cell polarity protein (FUZ) is regarded as a planar cell polarity effector that controls multiple cellular processes during vertebrate development 10 and an important regulatory gene that is expressed during *Drosophila melanogaster* development 11,12. Planar polarity signaling involves the interaction of multiple cellular behaviors, including cell proliferation 13,14. A recently published study revealed that exogenous FUZ expression markedly promoted cell proliferation of NSCLC cells and the upregulation of extracellular signal-regulated protein kinase 1/2 and signal transducer and activator of transcription 3 phosphorylation 15. Thus, we speculated that FUZ regulates the invasion and metastasis of lung cancer cells through the PI3K/AKT pathway, promotes the energy metabolism of cells, and increases the activity of the key enzymes and metabolites in glucose metabolism.

In this study, we investigated the effect of FUZ on NSCLC cells along with its mechanism of action.

## 2. Material and methods

### 2.1. Cell Culture and Transfection

The human lung adenocarcinoma cell lines A549, H460, H1299, PC9, HCC827, Calu1 and H292, as well as the human bronchial epithelial (HBE) cell line were obtained from the Chinese Academy of Sciences (Beijing, China). A549 cells were grown in Dulbecco's modified Eagle's medium (DMEM; HyClone, Logan, UT, USA), while H460, H1299, PC9, HCC827, Calu1, H292 and HBE cells were cultured in Roswell Park Memorial Institute 1640 medium (HyClone) supplemented with 10% fetal bovine serum (FBS; BI, Beit-Haemek, Israel) at 37 °C and 5% CO_2_. Lipofectamine 3000 (Invitrogen, Carlsbad, CA, USA) was used for transient transfection according to the manufacturer's protocol. Small interfering RNAs (siRNAs) were synthesized by Ribobio (Guangzhou, China) with the following sequences: siFUZ-1: 5′CCCUCAAUGGAGUCCACAUTT3′ (sense), 5′AUGUGGACUCCAUUGAGGTT3′ (antisense); siFUZ-2: 5′GGUCCUUCUUGUGGGACUUTT3′ (sense), 5′AAGUCCCACAAGAAGGACCTT3′ (antisense); and siFUZ-3: 5′UUCUCCGAACGUGUCACGUTT3′ (sense), 5′ACGUGACACGUUCGGAGAATT3′ (antisense). For FUZ overexpression, the cell lines were transfected with full-length FUZ complementary DNA, amplified by quantitative reverse transcription polymerase chain reaction (qRT-PCR), and cloned into the pcDNA3.1 vector to generate the pcDNA-FUZ construct. An empty pcDNA plasmid served as a negative control (NC).

### 2.2. Cell Proliferation Assay

Cells with a density of 3000 cells/well in 96-well plates were treated with various concentrations of gliclazide (0, 500, 1000, or 2000 μM) for 24, 48, 72, and 96 h. Thereafter, the cell viability assay (MTS assay; Promega, Madison, WI, USA) was carried out according to the manufacturer's instructions.

### 2.3. Cell Invasion and Migration Assay

For the transwell assay, 24 h after transfection, A549 or H1299 cells were serum-starved for 6 h. Next, 3 × 10^4^ cells (for A549 cells) or 5 × 10^4^ cells (for H1299 cells) in 200 μL serum-free medium were seeded into the upper chamber of a transwell plate with a fibronectin-coated filter (8 mm pore size; Corning Life Sciences, Corning, NY, USA). The lower chamber contained medium supplemented with 10% FBS. After 24 h of incubation at 37 °C, non-migrated cells were scraped off of the filter using a damp cotton swab. Following fixation with 4% paraformaldehyde, migrated cells were stained with crystal violet. The number of cells in six randomly chosen fields was counted. Each assay was performed in triplicate wells and repeated three times.

For the wound healing assay, H1299 and A549 cells were infected with NC/Plasmid-FUZ or transfected with si-NC/si-FUZ for 48 h and then transferred into serum-free medium. When the cell fusion reached 90%, the middle of each well was scratched with a standard 10 μL pipette tip. At each indicated time point, photographs of wound closure were taken using an inverted microscope (100× magnification; Nikon, Japan) and subsequently analyzed using ImageJ software V1.8.0.112 (National Institutes of Health, Bethesda, MD, USA).

### 2.4. qRT-PCR Analysis

Total RNA was isolated from cell lines using TRIzol reagent (Invitrogen, Carlsbad, CA, USA). RNA (800 ng) was reverse transcribed using the PrimeScript RT Reagent Kit with gDNA Eraser (Takara, Dalian, China), and qRT-PCR was performed using SYBR Premix Ex Tag II (Takara) in a qRT-PCR system with an annealing temperature of 60 °C. Actin was used as an internal control for normalization. The primer sequences used are listed in Table 1. Measurements were taken from three independent experiments.

### 2.5. Western Blot Analysis

After 48 h of transfection, cells were harvested and lysed in radioimmunoprecipitation buffer (Beyotime, Shanghai, China). After separation by 10% sodium dodecyl sulfate polyacrylamide gel electrophoresis, proteins were transferred to polyvinylidene fluoride membranes, blocked for 2 h in 5% skim milk, and incubated with antibodies at 4 °C overnight. Antibodies against FUZ, GLUT1, HK2, PKM2, LDHA, AKT, and phosphorylated (p)-AKT were obtained from Abcam (Cambridge, UK). β-actin and GAPDH was used as a loading control. An ECL Advanced Western Blot Detection Kit (Thermo Fisher Scientific, Waltham, MA, USA) was used to visualize specific protein bands, and Image Lab software V4.0 (Bio-Rad, Hercules, CA, USA) was used to analyze the western blot results. Antibodies used for western blot in this paper show in Table 2. All experiments were repeated three times.

### 2.6. ^18^F-fluorodeoxyglucose (^18^F‐FDG) Uptake Assay

Experiments were conducted using ^18^F‐FDG to assess the glucose uptake by A549 or H1299 cells, which were cultured in 6‐well cell plates until they reached approximately 80-90% confluence, and then, they were transfected with FUZ siRNA or FUZ overexpression plasmid. After 48 h, the cells were washed three times with phosphate-buffered saline (PBS) and incubated in 2 mL DMEM containing ^18^F‐FDG (148 kBq [4 μCi/mL]), for 1 h at 37 °C. Whole‐cell lysates were produced using 1 mL of trypsin‐ethylenediaminetetraacetic acid, and radioactivity was measured using a γ‐counter (ZonKia, Hefei, China). All tests were independently performed in triplicate.

### 2.7. Cell Metabolism Assay

To study changes in cellular metabolism, lactate levels were measured in the cell culture medium. Cells were seeded into 6‐well plates for transfection when they reached approximately 80-90% confluence. Then, 48 h after transfection with FUZ siRNA or FUZ overexpression plasmid, the culture medium was collected for the measurement of lactate concentrations. The tumor cells were washed, centrifuged, and lysed, and the lactate concentrations were measured using a Lactate Assay Kit (KeyGEN, Nanjing, China) according to the manufacturer's instructions. Optical densities were read at 530 nm using a microplate reader (Thermo Fisher Scientific, Waltham, MA, USA). Measurements were taken from three independent experiments.

### 2.8. Xenograft Studies and Micro-Positron Emission Tomography (PET) Scans

All *in vivo* experiments were performed in accordance with Institutional Review Board of China Medical University guidelines. Female athymic BALB/c nude mice (4-6-week-old) were purchased from Beijing Vital River Laboratory Animal Technology Co., Ltd. (animal experimental license no. SYXK2017-0033; Beijing, China). A549 cells (3 × 10^6^) in 100 μL PBS were subcutaneously injected into the right scapular region of the mice. Control siRNA or FUZ siRNA (Ribobio, Guangzhou, China) was injected twice a week after the average tumor size reached 30-50 mm^3^. Tumors were measured with a caliper every 2 days, and the body weight was also determined. Tumor volume was calculated using the formula V = 1/2 (width^2^ × length), and the metabolic levels of each tumor were relayed using Micro-PET scans (Madic, Shandong, China). Next, the mice were injected with a radiolabeled probe (250 μCi per mouse) via the lateral tail vein. After 60 min of uptake and image acquisition under 2% isoflurane anesthesia, static PET images were collected. The mice were terminated via CO_2_ inhalation when the tumor diameter reached 1.5 cm, according to the protocol filed with the Guidance of Institutional Animal Care and Use Committee of China Medical University.

### 2.9. Immunohistochemistry (IHC)

Tumors were formalin-fixed, paraffin-embedded, and prepared for staining with hematoxylin and eosin. Antibodies for KI67, FUZ, GLUT1, LDHA, AKT, and p-AKT were used for IHC. The staining was evaluated by scanning the entire tissue specimen under high magnification (20×). Protein expression was visualized and classified based on the percentage of positive cells and the intensity of staining. Five visual fields from each section were randomly selected.

### 2.10. Statistical Analysis

The results are presented as means ± standard deviations. Statistical analyses were conducted using the Student's* t*-test with SPSS Statistics version 22.0 (IBM SPSS, Armonk, NY, USA) and GraphPad Prism 8.0 (GraphPad Software, San Diego, CA, USA) software. *P* values < 0.05 were considered to be statistically significant. The results represent the mean of three replicate experiments conducted under the same conditions.

## 3. Results

### 3.1. FUZ is Overexpressed in NSCLC Cells

**Figure 1:**
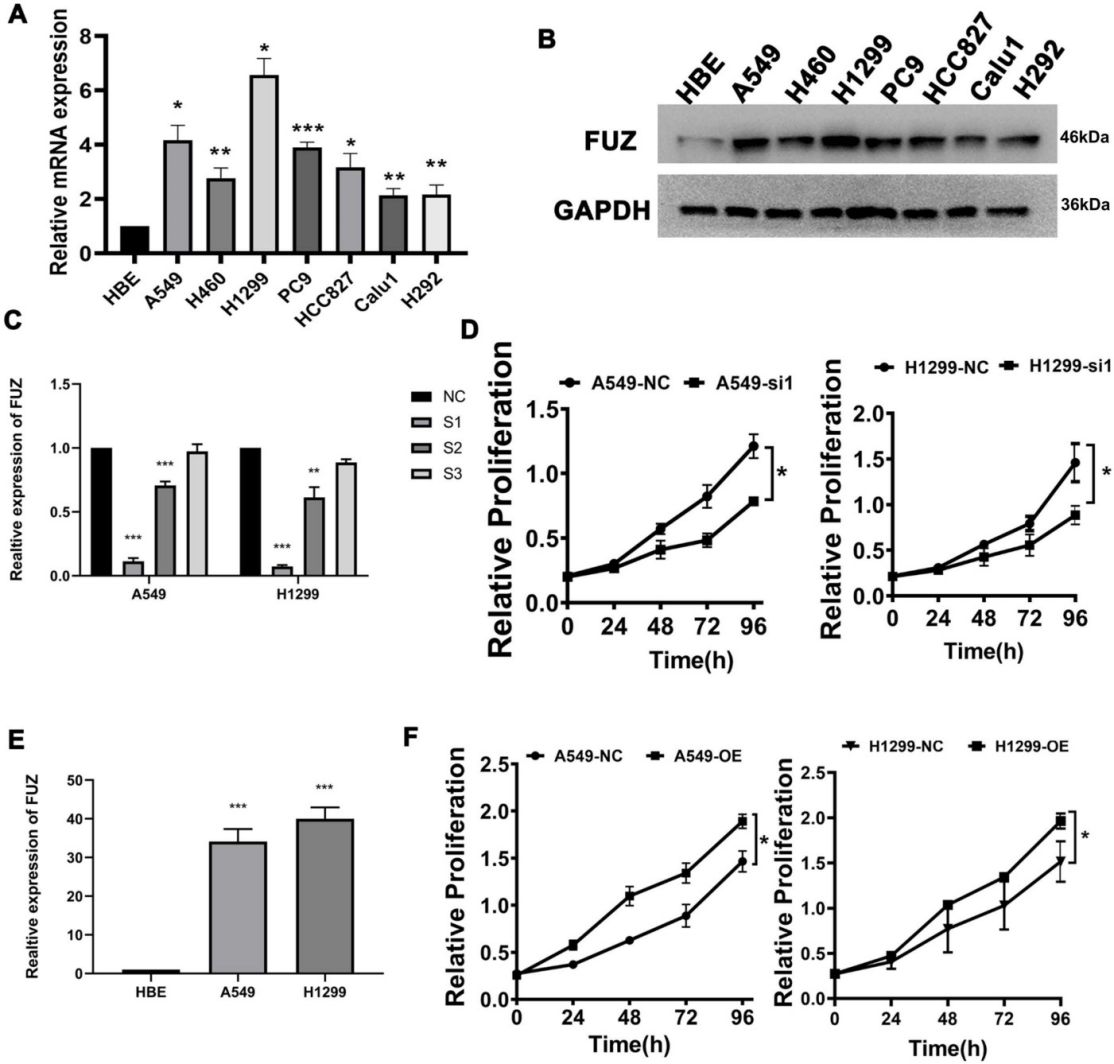
(A) Detection of FUZ expression by qRT-PCR. (B) Detection of FUZ expression using western blot analysis. (C, D) The expression of FUZ after 48 hours of transfection with NC and siRNA by qRT-PCR and the cell proliferation assay. S1, S2, and S3 are the three different siRNA sequences, and NC is negative control. (E, F) The expression of FUZ after 48 hours of transfection with NC and overexpression by qRT-PCR and the cell proliferation assay. NC is negative control. Measurements were taken from three independent experiments. *P < 0.05, **P < 0.01, and ***P < 0.001.

First, qRT-PCR and western blot analysis were conducted to assess FUZ expression in the NSCLC cell lines. The results demonstrated that FUZ was significantly upregulated in the NSCLC cell lines compared to that in the normal HBE cells and A549, H460, H1299, PC9, HCC827, Calu1 and H292 (Figure 1A). On comparison of FUZ expression levels among the normal control cells (HBE) and different NSCLC cells using western blot analysis, the results showed that FUZ was highly expressed in NSCLC cells, and we ultimately selected A549 and H1299 cells to determine the effects of FUZ knockdown and overexpression (Figure 1B). The expression of FUZ was lower in NSCLC cells transfected with siRNA (Figure 1C). Meanwhile, higher expression of FUZ was discovered in FUZ overexpression NSCLC cells (Figure 1E). The outcomes (Figure 1D,1F) were consistent with the CCK8 assay.

### 3.2. FUZ Promotes NSCLC Cell Invasion and Migration

**Figure 2:**
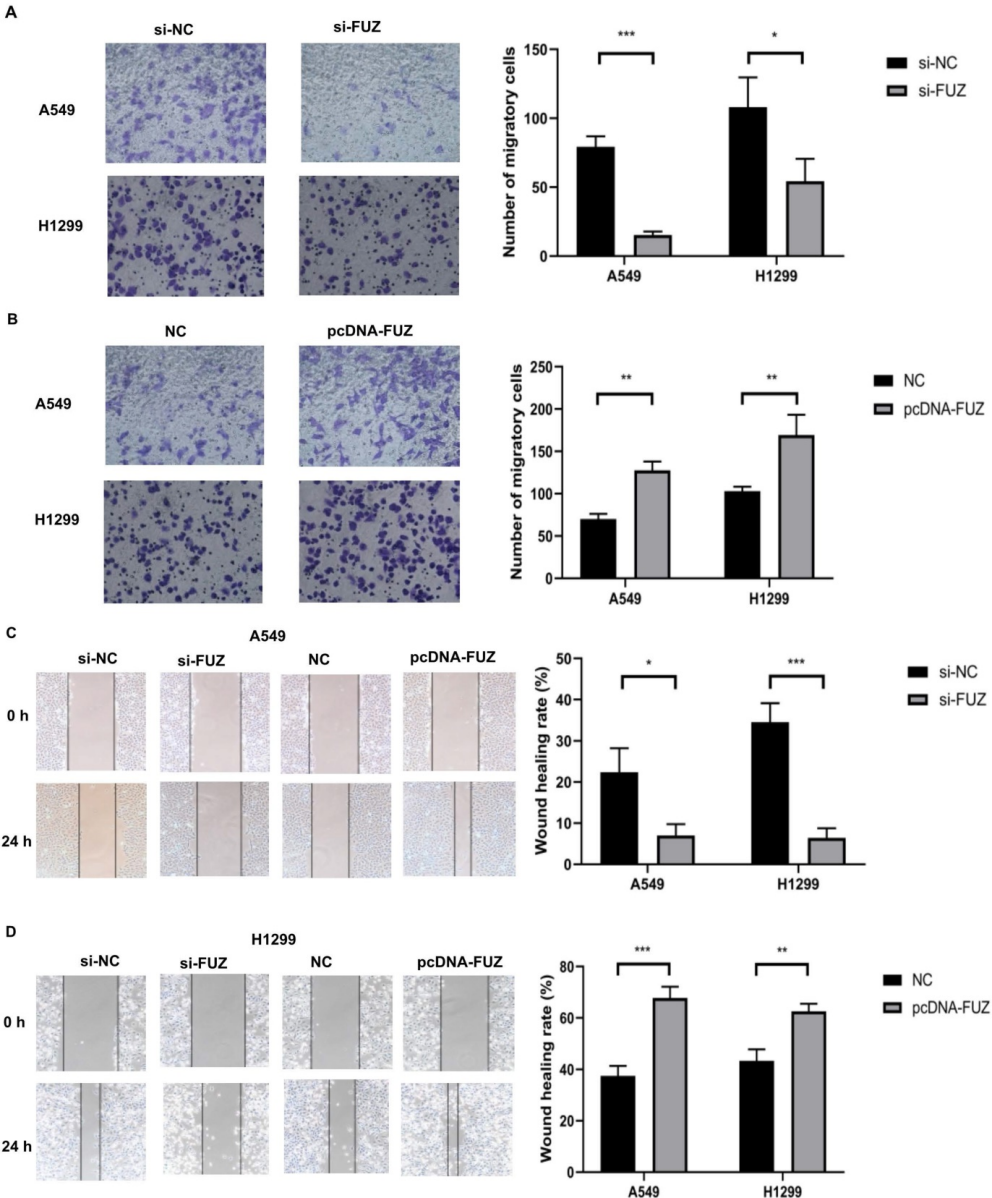
(A, B) The results of the transwell assays showing that FUZ enhances the invasion ability of NSCLC cells. (C, D) The results of the wound healing assays showing that FUZ promotes the migration ability of NSCLC cells. si-FUZ is FUZ siRNA, si-NC is siRNA negative control, pcDNA-FUZ is overexpressed FUZ, and NC is negative control. Measurements were taken from three independent experiments. *P < 0.05, **P < 0.01, and ***P < 0.001.

To determine whether FUZ can influence the invasion and migration of NSCLC cells, we transfected A549 and H1299 cells with plasmids overexpressing and siRNAs targeting FUZ. The cell invasion and migration capacities were evaluated using transwell assay and wound healing assay, respectively. The results demonstrated that FUZ knockdown significantly inhibited the invasion and metastasis of A549 and H1299 cells (Figure 2A, 2C and 2 D). Conversely, FUZ overexpression enhanced the invasion and metastasis capacities of A549 and H1299 cells (Figure 2B, 2C and 2D).

### 3.3. FUZ Promotes Glycometabolism in NSCLC Cells

**Figure 3:**
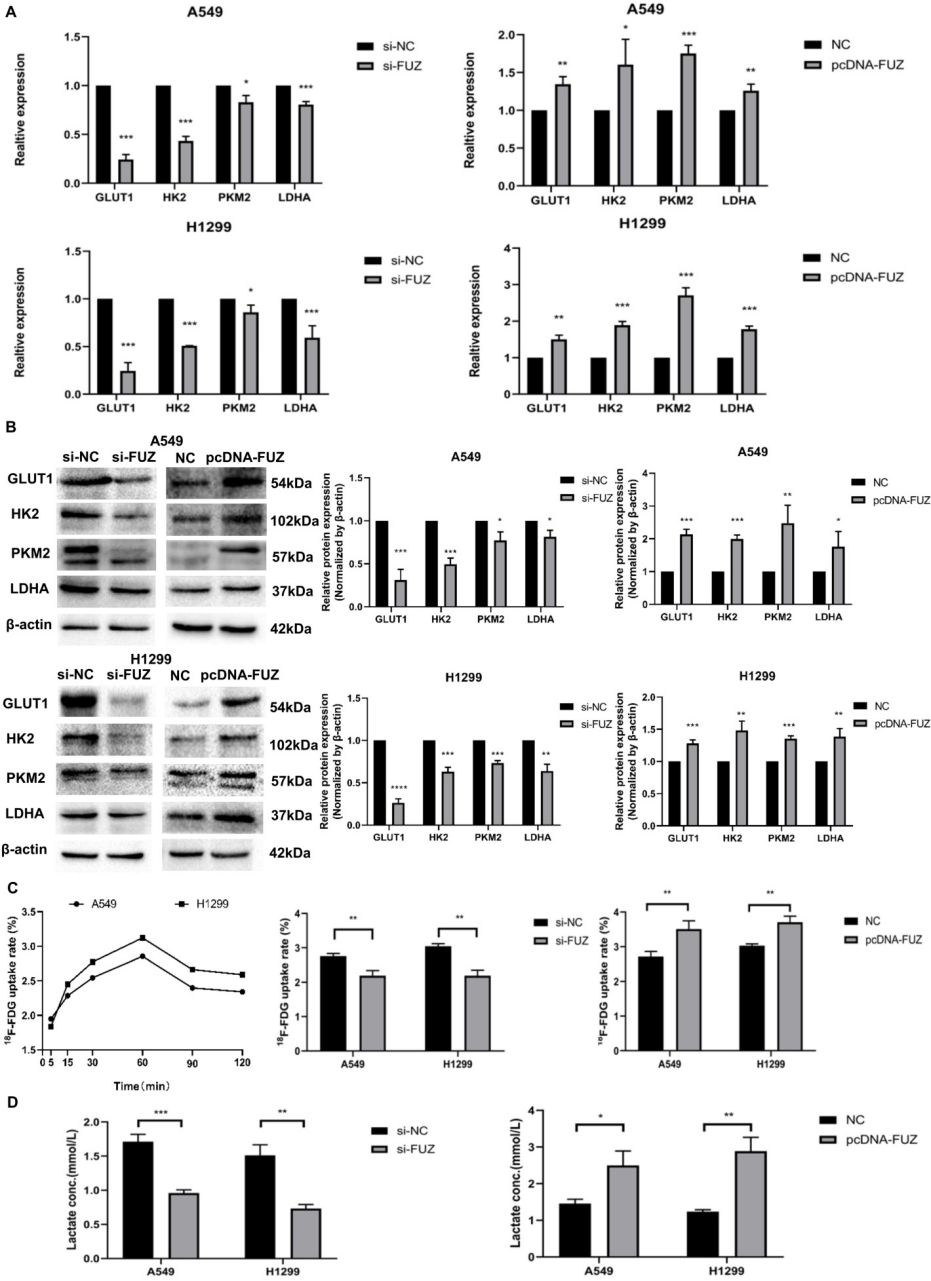
(A) Relative mRNA expression levels of t glycolysis related proteins s after FUZ knockdown and overexpression by qRT-PCR. (B) Relative expression levels of glycolysis related proteins after FUZ knockdown and overexpression using western blot analysis. (C) Cellular ^18^F‐FDG uptake was significantly decreased after FUZ siRNA transfection and increased upon FUZ overexpression. (D) Lactate levels in the culture medium of FUZ siRNA-transfected A549 and H1299 cells were significantly decreased, whereas they were increased upon FUZ overexpression. si-FUZ is FUZ siRNA, si-NC is siRNA negative control, pcDNA-FUZ is overexpressed FUZ, and NC is negative control. Measurements were taken from three independent experiments. *P < 0.05, **P < 0.01, and ***P < 0.001.

To elucidate the role of FUZ in glycometabolism, the expression levels of GLUT1, HK2, PKM2, and LDHA were examined using western blot and qRT-PCR analyses. The qRT-PCR results showed that the GLUT1, HK2, PKM2, and LDHA expression levels decreased after FUZ was downregulated, while the opposite was observed after FUZ was overexpressed (Figure 3A). Further, the GLUT1, HK2, PKM2, and LDHA protein levels were significantly decreased in both A549 and H1299 cells following FUZ downregulation, whereas they were increased upon FUZ upregulation (Figure 3B). To assess the effects of FUZ on glucose metabolism, cellular glucose uptake and lactic acid production were evaluated. ^18^F-FDG uptake, which was measured to evaluate the glucose uptake capacity of NSCLC cells, was decreased after FUZ siRNA transfection, while it was increased upon FUZ overexpression in both A549 and H1299 cells (Figure 3C). Cellular lactic acid levels in the culture medium were measured to assess changes in cellular metabolism. The results showed that lactic acid production was reduced in NSCLC cells transfected with FUZ siRNA, while it was elevated in NSCLC cells following FUZ overexpression (Figure 3D).

### 3.4. FUZ Promotes Glycometabolism via the PI3K/AKT Pathway in NSCLC Cells

**Figure 4:**
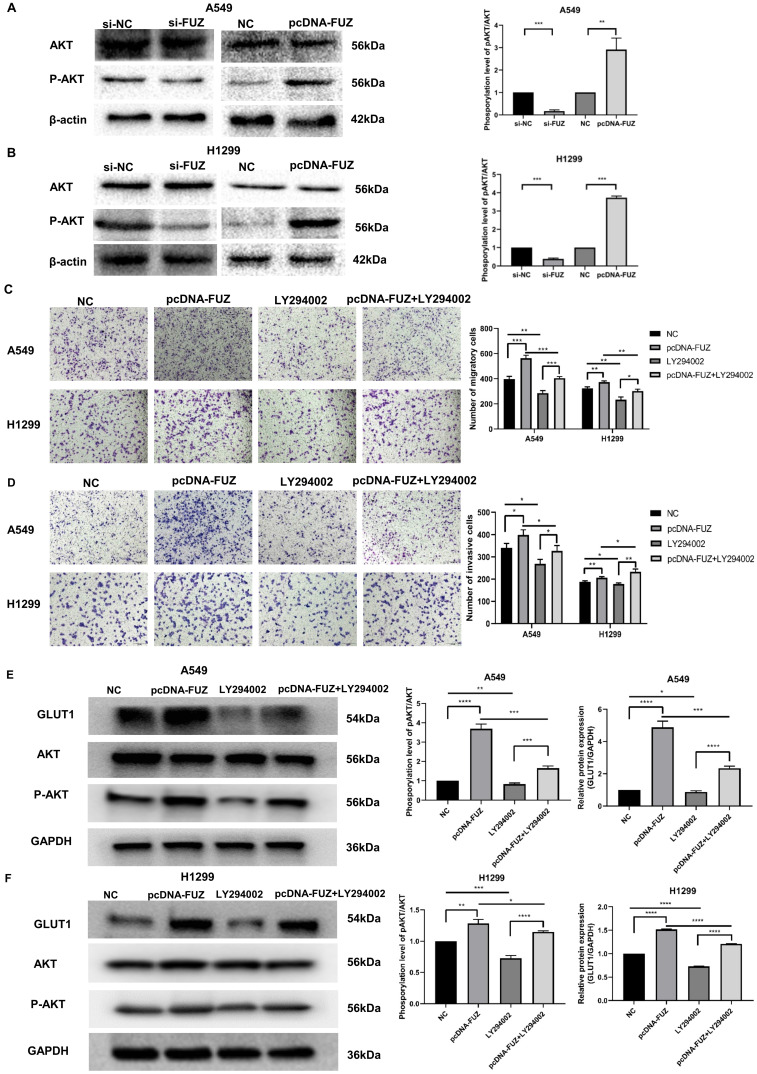
(A, B) The expression levels of AKT and p-AKT following FUZ knockdown and overexpression. si-FUZ is FUZ siRNA, si-NC is siRNA negative control, pcDNA-FUZ is overexpressed FUZ, and NC is negative control. (C) The results of transwell assay without Matrigel showing that FUZ enhances the migration ability were abolished by LY294002. (D) The results of transwell assay with Matrigel showing that FUZ enhances the invasion ability were abolished by LY294002. pcDNA-FUZ is overexpressed FUZ, LY294002 is PI3K inhibitor, pcDNA-FUZ+LY294002 is overexpressed FUZ+PI3K inhibitor, and NC is negative control. (E, F) The protein levels of GLUT1, AKT, and p-AKT, and were evaluated by the exposure of LY294002. Measurements were taken from three independent experiments. *P < 0.05, **P < 0.01, and ***P < 0.001.

PI3K/AKT is an important pathway that affects tumor energy metabolism and alters the biological behavior of tumors. To investigate whether FUZ can regulate glucose metabolism, cell migration, and cell invasion in tumor cells via PI3K/AKT signaling, the expression levels of AKT and p-AKT were evaluated by western blot analysis. Knockdown of FUZ clearly reduced p-AKT levels in both A549 and H1299 cells. Consistent with these findings, FUZ overexpression increased p-AKT levels (Figure 4A and 4B). In addition, we cultured NSCLC cells with a PI3K inhibitor (LY294002). The results of transwell assay without Matrigel demonstrated that suppression of the PI3K/AKT signaling pathways counteracted the role of FUZ overexpression in promoting migration in both A549 and H1299 cells (Figure 4C). The results of transwell assay with Matrigel demonstrated that suppression of the PI3K/AKT signaling pathways counteracted the role of FUZ overexpression in promoting invasion in both A549 and H1299 cells (Figure 4D). Moreover, the results of western blot analysis demonstrated that suppression of the PI3K/AKT signaling pathways counteracted the role of FUZ overexpression in promoting the expression of GLUT1 and p-AKT levels in both A549 and H1299 cells (Figure 4E and 4F). These findings show that FUZ is involved in cell migration and cell invasion via the PI3K/AKT signaling pathway and suggest that FUZ may regulate key enzymes in the glucose metabolism pathway via PI3K/AKT signaling to promote glucose metabolism in NSCLC.

### 3.5. si-FUZ Inhibits NSCLC Tumor Growth and Glycometabolism In Vivo

**Figure 5:**
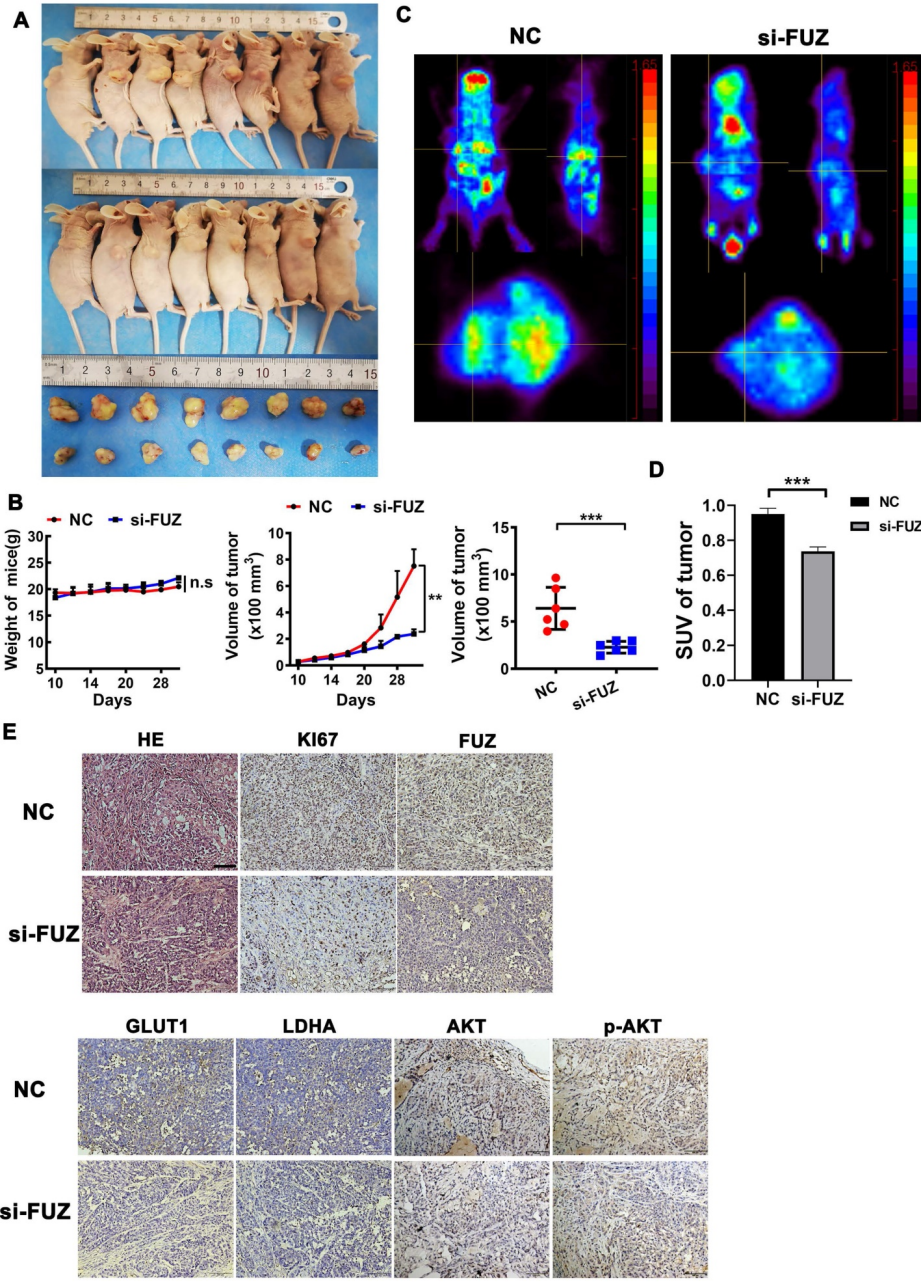
(A) Tumor sizes in the si-FUZ and NC groups. (B) The mice weight and tumor volume curves of the si-FUZ and NC groups. Tumor volumes in the si-FUZ group were significantly decreased compared to those in the NC group. (C) Representative micro-PET images of mice in the si-FUZ and NC groups. (D) ^18^F-FDG SUVmax values in the si-FUZ group were significantly decreased compared to those in the NC group. (E) Representative IHC images of KI67, FUZ, GLUT1, LDHA, AKT, and p-AKT staining in A549 xenografts. si-FUZ is FUZ siRNA and NC is negative control. Measurements were taken from three independent experiments. ***P < 0.001.

To explore the function of FUZ in NSCLC tumor growth *in vivo*, a xenograft model was constructed. si-FUZ significantly inhibited tumor proliferation in the xenograft model (Figure 5A). The tumor volumes in the si-FUZ group were smaller than those in the NC group (Figure 5B). To investigate whether FUZ can regulate tumor glycometabolism levels *in vivo*, micro-PET scans were performed, which revealed that after treatment with si-FUZ, the ^18^F-FDG maximum standardized uptake values (SUVmax) of the tumors were significantly decreased (Figure 5C and 5D). IHC analysis showed a significant decrease in KI67, FUZ, GLUT1, LDHA, AKT, and p-AKT levels in the si-FUZ group (Figure 5E).

## 4. Discussion

The incidence and mortality of lung cancer in China continue to increase annually 16. In recent years, multiple new oncogenes and tumor suppressors have been successfully identified, which has improved the understanding of tumorigenesis and tumor progression. However, the clinical manifestations of lung cancer are complex and individualized. Furthermore, there is no effective chemotherapy for NSCLC. Although some progress has been made in the diagnosis and treatment of NSCLC, the overall prognosis of patients remains poor 17,18. Therefore, it is important to study the mechanism of NSCLC progression and explore novel therapeutic targets. With the accumulation of tumor-related gene knowledge, additional tumor markers have been identified for the therapy and outcomes of NSCLC. The discovery of these potential tumor markers is important for clarifying the mechanisms of tumorigenesis as well as for assisting in diagnosis and treatment.

Extensive research has been conducted on the role of FUZ in the nervous system, and recent studies have found that FUZ also plays an important role in tumor development. Chen et al. revealed that FUZ expression is associated with patient survival probabilities in eight types of cancers 19. He et al. demonstrated that FUZ is associated with the poor prognosis of NSCLC patients. In addition, FUZ promotes NSCLC proliferation and the epithelial-mesenchymal transition process *in vitro* 15. The present study focused on the biological role and mechanism of FUZ in promoting NSCLC energy metabolism, migration, and invasion via the PI3K/AKT pathway.

According to our research findings, FUZ promoted cell proliferation *in vitro* (Figure 1D and 1F). In addition, FUZ expression was upregulated in the NSCLC cell lines, as per the results of the qRT-PCR analysis (Figure 1C and 1E). Thus, the results suggest that FUZ might promote the proliferation of NSCLC cells. Next, we performed transwell and wound healing assays to determine the effect of FUZ on the NSCLC cell migration and invasion capacities, respectively. The results showed that FUZ knockdown significantly inhibited the migration and invasion of A549 and H1299 cells. Conversely, FUZ overexpression enhanced the migration and invasion capacities of A549 and H1299 cells (Figure 2A and 2B).

Here, we demonstrated for the first time that FUZ expression is positively correlated with that of GLUT1, HK2, PKM2, and LDHA in NSCLC (Figure 3A and 3B). Previous studies have shown that one mechanism underlying the increase in glycometabolism in malignant tumor cells is the overexpression of glucose transporters, with high GLUT1 expression detected in NSCLC 20,21. Moreover, HK2 and PKM2 are rate-limiting enzymes that catalyze key steps in cellular glycometabolism 22,23, and LDHA is another important enzyme for glycolysis 7. Changes in these key enzymes can reflect glycometabolism alterations in NSCLC. Hence, our data indicate that FUZ can promote glycometabolism by inducing GLUT1, HK2, PKM2, and LDHA overexpression in NSCLC.

The PI3K/AKT pathway has an important function in the glycometabolic reprogramming of tumor cells. AKT, also known as the Warburg kinase, is a core factor in the PI3K/AKT pathway 24, 25. Our results revealed that p-AKT levels were increased in both A549 and H1299 cells after FUZ knockdown, with the opposite effect observed upon FUZ overexpression (Figure 4A). Therefore, we speculated that FUZ induces activation of the PI3K/AKT pathway and promotes GLUT1, HK2, PKM2, and LDHA expression, resulting in an increased glucose metabolism rate.

Changes in tumor metabolism can be monitored *in vivo* using molecular imaging methods, such as PET. By labeling specific molecules in tumor metabolism with radionuclides, PET can demonstrate the molecular mechanism of tumor metabolism *in vivo*. ^18^F-FDG is the most commonly used radiotracer in clinical practice. Tumors tend to undergo aerobic glycolysis based on the Warburg effect 26, which appears as an abnormal accumulation of ^18^F-FDG. PET imaging plays an important role in many tumor aspects 27. In this study, we used micro-PET scans with ^18^F-FDG and found that after treatment with si-FUZ, the ^18^F-FDG SUVmax values of the tumors were significantly decreased (Figure 5C). Furthermore, this trend was also validated by the IHC results (Figure 5E), implying that if FUZ is used as a potential therapeutic target, ^18^F-FDG PET scans can effectively identify the therapeutic response. Experiments using tissue samples are required to confirm the physiological relevance of this findings in the future.

## 5. Conclusion

In summary, FUZ exhibits a significant association with glycometabolism, regulates cell invasion and metastasis, and promotes ^18^F-FDG uptake and lactate production via the PI3K/AKT pathway in NSCLC. Therefore, our research indicates that FUZ may be a potential therapeutic target that can be beneficial in the diagnosis and treatment of NSCLC. In addition, using PET imaging to dynamically monitor therapeutic efficacy may be a feasible model for NSCLC tumor management.

## Figures and Tables

**Table 1:** Sequences of primers for qRT-PCR.

Gene	Forward (5′-3′)	Reverse (5′-3′)
FUZ	CCCUCAAUGGAGUCCACAUTT	AUGUGGACUCCAUUGAGGTT
GLUT1	CTGGCATCAACGCTGTCTTC	GCCTATGAGGTGCTGGGTC
HK2	GAGCCACCACTCACCCTACT	ACCCAAAGCACACGGAAGTT
PKM2	GGGTTCGGAGGTTTGATG	ACGGCGGTGGCTTCTGT
LDHA	TTCACCCCCAGGAACTC	ATCCCGTGTCCGAAGGA

**Table 2:** Antibodies used for western blot in the present study.

Antibody name	Source	Catalog number
Anti-FUZ	Abcam	ab111842
Actin	Abcam	ab8226
GLUT1	Abcam	ab115730
LDHA	Abcam	ab52488
PKM2	Abcam	ab85555
HK2	Abcam	ab209847
GAPDH	Abcam	ab8245
AKT	Abcam	ab38449
P-AKT	Abcam	ab8805

## Abbreviations

qRT-PCR: Quantitative real-time polymerase chain reaction
^18^F-FDG: 18 Fluorine fluorodeoxyglucose
NSCLC: Non-small cell lung cancer
HK2: Hexokinase 2
PKM2: Pyruvate kinase 2
LDHA: Lactate dehydrogenase A
PCP: Planar cell polarity
HBE: Human bronchial epithelial
NC: Negative control
IHC: Immunohistochemical
